# Antistaphylococcal Activity of Extracts, Fractions, and Compounds of *Acacia polyacantha* Wild (Fabaceae)

**DOI:** 10.1155/2020/2654247

**Published:** 2020-03-16

**Authors:** Fred A. Ashu, Jean Na-Iya, Brice E. N. Wamba, Justin Kamga, Paul Nayim, Bathélémy Ngameni, Veronique P. Beng, Bonaventure T. Ngadjui, Victor Kuete

**Affiliations:** ^1^Department of Biochemistry, Faculty of Science, University of Dschang, Dschang, Cameroon; ^2^Department of Biochemistry, Faculty of Science, University of Yaoundé I, Yaoundé, Cameroon; ^3^Department of Organic Chemistry, Faculty of Science, University of Yaoundé I, Yaoundé, Cameroon; ^4^Department of Pharmacognosy and Pharmaceutical Chemistry, Faculty of Medicine and Biomedical Science, University of Yaoundé I, Yaoundé, Cameroon

## Abstract

*Acacia polyacantha* is a medicinal plant traditionally used to treat livestock diseases and gastrointestinal infections; our study was undertaken to evaluate the antistaphylococcal activities of the methanolic leaf, bark, and root extracts, fractions, and compounds from *Acacia polyacantha* against a panel of 14 multidrug-resistant *Staphylococcus* bacterial strains overexpressing efflux pumps. The study was also extended to investigate two possible modes of action, that is, influence on bacterial growth kinetics and influence on proton-ATPase pumps, of the most active compound against a reference strain. *Materials and Methods*. The crude extracts after extraction were subjected to column chromatography. Antibacterial assays of extracts, fractions, and compounds alone and in the presence of efflux pump inhibitors were carried out using the broth microdilution method and the study of two mechanisms of action achieved by standard methods with the most active compound. *Results*. The phytochemical study of *Acacia polyacantha* leaves leads to the isolation of stigmasterol (**1**), *β*-amyrin (**2**), 3-*O*-methyl-_D_-chiro-inositol (**3**), epicatechin (**4**), quercetin-3-*O*-galactoside (**5**), 3-*O*-[*β*-_D_-xylopyranosyl-(1 ⟶ 4)-*β*-_D_-galactopyranosyl]-oleanolic acid (**6**), 3-*O*-[*β*-galactopyranosyl-(1⟶ 4)-*β*-_D_-galactopyranosyl]-oleanolic acid (**7**) and that of leaves lead to the isolation of lupeol (**8**) 2,3-dihydroxypropyltetracosanoate (**9),** and methyl-gallate (**10**). Leaf, root, and bark extracts inhibited 92.85% (13/14), 92.85% (13/14), and 71.43 % (10/14) of the tested bacteria strains, respectively, with minimum inhibitory concentration (MIC) varying between 16 and 1024 *μ*g/mL. Fractions exhibited better activities compared to those of their extracts of origin, as their MICs ranged from 16 to 512 *μ*g/mL, with fractions from leaves being more active than those obtained from barks. Compounds had varying activities; MICs varied from 16 to 512 *μ*g/mL with compound **4** presenting the best activity as MICs ≤100 *μ*g/mL were obtained against 11 of the tested bacteria. The activities of extracts, fractions, and compounds were improved in the presence of carbonyl cyanide m-chlorophenylhydrazone (CCCP) as an efflux pump inhibitor to as much as >128 folds. Meanwhile, in the presence of chlorpromazine as an efflux pump inhibitor, only the activity of compound **10** was improved on 10 of the tested bacteria strains. Compound **4** prolonged the lag phase of the growth kinetic in a concentration-dependent manner and equally inhibited the proton-ATPase pumps of the tested bacteria strains. *Conclusion*. The present study demonstrates the antistaphylococcal potential of *Acacia polyacantha* and its constituents to combat bacterial infections alone or in combination with efflux pump inhibitors.

## 1. Introduction

Bacterial infections are a major burden to the public health sector, as they are responsible for an estimated 560,000 deaths yearly worldwide [1]. This is further complicated by the alarming rate of the emergence of drug-resistant strains [2]. Among these infections, those caused by *Staphylococcus aureus* are globally responsible for 7–10% of deaths annually [3]. *Staphylococci* are responsible for a wide range of community and nosocomial infections such as septicemia, endocarditis, and cutaneous infections [4]. The fight against *Staphylococci* infections has been a challenging one since multidrug-resistance emerged; this phenomenon has been mostly attributed in the case of Gram-positive bacteria to the overexpression of efflux pumps and presence of antibiotic-degrading enzymes [5]. This phenomenon of resistance propels the search for new antimicrobial agents with higher efficacy and low toxicity [6]. Plants and their secondary metabolites have long been used by humans in the treatment of ailments caused by these pathogens [7]. According to the World Health Organization, 65% of the world's population have integrated the use of medicinal plants as therapy in the primary modality for healthcare, and in Africa, 80% of individuals use some sort of traditional herbal medicine [8].

Cameroon's flora constitutes a prominent reservoir of secondary metabolites with potential antimicrobial activity; among these are those previously reported such as *Treculia obovoidea* (Moraceae) [5], *Piper guineense* (Piperaceae), *Fagara leprieurii* (Rutaceae), and *Monodora myristica* (Annonaceae) [9]. In order to curb the phenomenon of resistance, more secondary metabolites of plants need to be studied. The present study was designed to evaluate the antistaphylococcal potential of extracts, fractions, and compounds from *Acacia polyacantha* against fourteen strains of *S. aureus* and to investigate two modes of action of the most active compound. *Acacia polyacantha* is a deciduous, straight cylindrical, erect tree of about 10–15 m height found in Tropical Africa, occurring from Gambia to Ethiopia and southwards to Kenya and Zimbabwe [10, 11]. The root extract is used as a treatment for snake bites and applied to wash the skin of children who are agitated at nighttime [12]. The root extracts and possibly the bark extracts are used in the treatment of venereal diseases, dysentery, and gastrointestinal disorders [13]. Infusion of stem bark is used to treat jaundice, while powdered root mixed with honey is used for cough and asthma [14]. Previous phytochemical investigations of the leaves of the plant led to the isolation of polyacanthoside A, oleanolic acid, stigmasterol, stigmasterol-3-*O*-*β*-glucopyranosyl, epicatechin, quercetin-3-*O*-glucoside,3-*O*-methyl-_D_-chiro-inositol, 3-*O*-[*β*-_D_-galactopyranosyl-(1 ⟶ 4)-*β*-_D_-galactopyranosyl]-oleanolic acid quercetin-3-*O*-glucoside, and 3-*O*-[*β*-_D_-xylopyranosyl-(1 ⟶ 4)-*β*-_D_-galactopyranosyl]-oleanolic acid [11]. The present study was thus aimed at evaluating the antistaphylococcal activity of extracts, fractions, and compounds from *Acacia polyacantha* against multidrug-resistant *Staphylococcus* species. The study was also extended to the investigation of two modes of action of the most active compound such as influence on the growth kinetics and on the proton-ATPase pumps.

## 2. Materials and Methods

### 2.1. General Procedure

Horiba SEPA-300 polarimeter (HORIBA, Kyoto, Japan) was used to measure the optical rotation. NMR spectra were recorded on Bruker DMX Avance 600 instruments equipped with an autotune probe and using the automation mode aided by the Bruker program. HREI-SMS spectra were determined on a micrOTOF-Q 98 spectrometer. For column chromatography, silica gel 60 particle sizes 0.04–0.063 mm (Merck) and Sephadex LH-20 (Sigma) were used. The plates were visualized using UV (254 and 366 nm) and revealed by spraying with vanillin-sulphuric acid.

### 2.2. Plant Material

The plant materials (leaves, stem barks, and roots) were collected in March 2017 in the Kaele locality of the north region of Cameroon and subsequently identified at the national herbarium of Cameroon by Mr. Victor Nana, where voucher specimens were deposited under the identification number 58985/SRF/CAM. They were air-dried. Finely powdered leaves (2 kg), barks (2.5 kg), and roots (1.5 kg) of *Acacia polyacantha* were each subjected to extraction by maceration method, twice with 4 L, 7.5 L, and 5 L of methanol (MeOH), respectively, for 48 hours. The solvent of each solution was evaporated under reduced pressure to give 225 g, 135.59 g, and 25 g of total crude extract of leaves, stem barks, and roots, respectively. The extracts were stored at 4°C for further use. The yields of leaves (225 g) were 11.25%, the extract of stem barks (135.59 g) gives a percentage of 5.42%, and for roots (25 g), we obtained 1.66%.

### 2.3. Isolation from Leaves of *Acacia polyacantha*

The crude extract (225 g) of leaves of *Acacia polyacantha* was dissolved in a mixture of petroleum ether/ethyl acetate (99 : 1) and shaken to remove a dark green extract of chlorophyll. The residue (110 g) was subjected to silica gel column chromatography (40–63 *μ*m, 6 × 50 cm) using *n*-hexane-AcOEt and CHCl_3_-MeOH gradients as eluents. 198 fractions of 300 ml each were collected as follows: [(1–13), *n*-hexane-AcOEt95 : 5], [(14–29), *n*-hexane-AcOEt90 : 10], [(30–63), *n*-hexane-AcOEt85 : 15], [(64–117), *n*-hexane-AcOEt80 : 20], [(118–122), *n*-hexane-AcOEt70 : 30], [(123–129), *n*-hexane-AcOEt60 : 40], [(130–140), CHCl_3_-MeOH97.5 : 2.5], [(141–152), CHCl_3_-MeOH 95 : 5], [(153–166), CHCl_3_-MeOH 80 : 10], [(167–182), CHCl_3_-MeOH 85 : 15], [(183–190), CHCl_3_-MeOH 80 : 20], and [(191–198), CHCl_3_-MeOH 75 : 25]. These fractions were combined on the basis of their TLC profiles to give 13 subfractions (*F*_1_′-*F*_13_′) as follows: *F*_1_′ (1–14, 8.5 g), *F*_2_′ (15–20,6.3 g), *F*_3_′ (21–27, 11.8 g), *F*_4_′ (28–68, 13.5 g), *F*_5_′ (69–95, 10.6 g), *F*_6_′ (96–133, 10.2 g), *F*_7_′ (134–154, 7.5 g), *F*_8_′ (155–161, 6.2 g), *F*_9_′ (162–171, 6.4 g), *F*_10_′ (172–183, 7.3 g), *F*_11_′ (184–187, 4.4 g), *F*_12_′ (188, 2.2 g), and *F*_13_′ (189–198, 6.8 g). Subfraction *F*_9_′ was purified with Sephadex LH-20 using isocratic CHCl_3_-MeOH (7 : 3) as eluent. Fractions of 5 mL were collected. From subfractions *F*_9_′6–11 of this column, compound **3** (18 mg) was obtained as a white powder while compound **4** (95.2 mg) was isolated from fractions 35–50 of the same column as a red powder. Compounds **1** (44.1 mg) and **2** (45.9 mg) were obtained, respectively, as white powders directly by simple washing and filtration from *F*_1_′ and *F*_3_′. Subfraction *F*_11_′ was purified twice by Sephadex LH-20 using CHCl_3_-MeOH (7 : 3), the solvent system from which compound **5** (2.65 g) was obtained as a yellow powder. Subfractions *F*_12_′ and *F*_13_′ from leaves were purified twice by Sephadex LH-20 using the same solvent system, from where compounds **6** (25 mg) and **7** (10.2 mg) were obtained, respectively, as white powders. The yield of leaves was 11.25 %.

### 2.4. Isolation from the Stem Bark of *Acacia polyacantha*

The stem bark crude extract (135.59 g) of *Acacia polyacantha* was partitioned between AcOEt (750 mL × 3) and *n*-butanol (750 mL × 3) to give 36.2 g of AcOEt and 54.7 g of *n*-BuOH fractions. The ethyl acetate fraction (36.2 g) was adsorbed on an equivalent mass of silica and chromatographed over a silica gel column chromatography (40–63 *μ*m, 4.5 × 50 cm) using *n*-hexane-AcOEt and CHCl_3_-MeOH gradients as eluents. Fractions of 250 mL each were collected as follows: [(1–3), *n*-hexane 100%], [(4–8), *n*-hexane-AcOEt 5%] [(9–15), *n*-hexane-AcOEt 10%], [(16–33), *n*-hexane-AcOEt 15%], [(34–46), *n*-hexane-AcOEt 20%], [(47–57), *n*-hexane-AcOEt 30%], [(58–60), *n*-hexane-AcOEt 50%], [(61–63), CHCl_3_-MeOH 5%], [(64–70), CHCl_3_-MeOH 10%], and [(71–85), CHCl_3_-MeOH 15%]. These fractions were combined on the basis of their TLC profiles to give 13 subfractions (*F*_1_-*F*_12_) as follows: *F*_1_ (1–5, 5.3 g), *F*_2_ (6–8,4.2 g), *F*_3_ (9–14, 4.3 g), *F*_4_ (15–24, 1.1 g), *F*_5_ (25–32, 2.8 g), *F*_6_(33–42, 2.2 g), *F*_7_ (43–49, 1.5 g), *F*_8_ (50–55, 2.7 g), *F*_9_ (56–64, 2.9 g), *F*_10_ (65–69, 1.5 g), *F*_11_ (70–75, 2.4 g), and *F*_12_ (76–85, 4.1 g). Compound **8** (123.2 mg) was isolated as white crystal by filtration of the precipitates of the subfractions *F*_2_. Compound **9** (12 mg) was isolated as a white powder by filtration of the precipitates of the subfractions *F*_7_. From *F*_8_, compound **10** (10.5 mg) was obtained as a white powder. This extract (135.5 g) gave a yield percentage of 5.42%.

### 2.5. Antibacterial Assay

#### 2.5.1. Chemicals

The chemicals used in this study included a reference antibiotic ciprofloxacin (CIP), dimethyl sulfoxide (DMSO) used to solubilize plant extracts, fractions, and compounds, *p*-iodonitrotetrazolium chloride (INT) used as microbial growth revelator, and carbonyl cyanide *m*-chlorophenylhydrazone (CCCP) and chlorpromazine (CPZ) (Sigma-Aldrich) used as efflux pump inhibitors (EPIs).

#### 2.5.2. Bacteria, Culture Media, and Growth Condition

The strains of *Staphylococcus aureus* used included a reference strain obtained from the American Type Culture Collection (ATCC; ATCC 25923), seven methicillin-resistant *S. aureus* (MRSA) strains (MSSA1, MRSA3, MRSA4, MRSA6, MRSA8, MRSA9, MRSA11, and MRSA12) (obtained from the culture collection of the Laboratory of Microbiology, Graduate School of Pharmaceutical Sciences, the University of Tokyo, Japan, and provided by Dr. Jean P. Dzoyem, University of Dschang) [15, 16], and five resistant clinical laboratory strains (SA01, SA07, SA18, SA23, and SA88) available in our laboratory collection and previously isolated from patients in Ad-Lucem Hospital in Banka-Bafang (West Region of Cameroon) [17]; their features were previously reported [5]. These bacterial strains were cultured on Mueller–Hilton Agar (MHA) 24 hours prior to susceptibility testing; meanwhile, Mueller–Hilton Broth (MHB) was used during the assay [5].

#### 2.5.3. INT Colorimetric Assay for MIC and MBC Determinations

The determination of minimum inhibitory concentration (MIC) and minimum bactericidal concentrations (MBCs) was carried out through the rapid INT colorimetric assay [18] with some slight modifications [19]. DMSO/MHB was used to solubilize the plant extracts, fractions, compounds as well as reference antibiotics, with the final concentration of DMSO less than 2.5%, which does not affect bacterial growth. The solution obtained was then added to the wells of a 96-well microtiter plate containing MHB and serially diluted in twofold. One hundred microliters of inoculum (1.5 × 10^6^ CFU/mL) prepared in the appropriate broth was then added. These plates were sealed and the MIC of each sample was detected after 18 h of incubation at 37°C, upon addition (40 *μ*L) of 0.2 mg/mL of INT, and incubation at 37°C for 30 minutes as the least concentration of the sample that completely inhibited microbial growth and, thus, prevented the color change of the medium. The MBCs were determined by introducing 150 *μ*L of broth culture into the wells of new plates, and then the volume was topped up to 200 *μ*L by adding 50 *μ*L of the contents of wells with a concentration greater than or equal to the MIC. These plates were then incubated for 48 hours at 37°C followed by a revelation at INT.

Each assay was performed in three independent tests and in triplicate. MIC and MBC were valid if at least two identical values were obtained

#### 2.5.4. Evaluation of the Antibacterial Activity of Different Extracts, Fractions, Products, and Antibiotics in the Presence of Inhibitors

To access the role played by efflux pumps in the resistance of the bacteria strains, the activity of extracts, some fractions, and compounds was evaluated in the absence and presence of two efflux pump inhibitors: chlorpromazine (25 *μ*g/mL) and carbonyl cyanide *m*-chlorophenylhydrazine (0.5 *μ*g/mL). The MICs of samples alone and in the presence of EPIs were determined just as described above and the activity improvement factor was calculated as the ratio of MICs of samples alone to that in the presence of EPIs. These assays were carried out in triplicate.

After the evaluation of the antistaphylococcal activity of the plant samples, the most active compound **4** on the reference strain (ATCC25923) was retained and two modes of action were investigated: influence on the bacterial growth kinetics and ATPase-proton pumps.

### 2.6. Influence on Growth Kinetics

This was carried out to determine during which phase of the growth kinetics the most active substance exerts its action and to equally confirm if the effect of the most active compound is bacteriostatic or bactericidal. The protocol used was previously described by Cox et al. [20] with slight modifications. Briefly, bacterial suspensions of 1.5 × 10^8^ CFU/mL were prepared from 18-hour-old cultures. These suspensions were diluted with MHB to obtain 22 mL of inoculum with a concentration of approximately 10^6^ CFU/mL. Test compound (0.5 mL) at concentration of MIC/2, MIC, and 2 × MIC was then introduced, and the different tubes were incubated at 37°C for varying time intervals (0, 0.5, 1, 2, 4, 6 hrs,…); at the end of each interval, 500 *μ*L of the content from these tubes was collected and the optical density was read at 600 nm. Negative control was made up of tubes containing MHB, inoculum, and DMSO. The results permitted us to plot a graph of optical density against time.

### 2.7. Influence on Proton-ATPase Pumps

This was carried out by the follow-up of glucose-induced acidification of the medium. Briefly, few colonies of bacteria were collected from a previously cultured Petri dish and introduced into 20 mL of MHB and then incubated at 37°C for 18 hours after which aliquots were collected and diluted at 1/100 v/v in a conical flask. 100 mL of bacterial cells was centrifuged at 4000 rpm for 30 minutes at 4°C; pellets obtained were successively washed with distilled water and 50 mM potassium chloride (KCl) solution and resuspended in 50 mL of 50 mM of the same solution after which they were conserved at 4°C for 18 hours. The pH of the solutions was then adjusted to 6.4 by adding moderate quantities of NaOH and/or HCl. To 4 mL of these solutions was added 0.5 mL of the test compound dissolved in DMSO to obtain concentrations corresponding to 2 × MIC, MIC, and MIC/2. After 10 minutes of preincubation at 37°C, the acidification of the medium was initiated by the addition of 0.5 mL of glucose solution (20%) whose catabolism is accompanied by the liberation of protons into the medium. Finally, the pH of the medium was measured every 10 minutes for 1 hour. As for the negative control, the compound solution was replaced by DMSO. The values of pH measured permitted us to plot a graph of variation of pH as a function of time. All inhibition of the acidification of the medium in the presence of a compound was attributed to the inhibition of the proton-ATPase pumps by the compound [21].

## 3. Results

### 3.1. Phytochemistry

**Figure 1:**
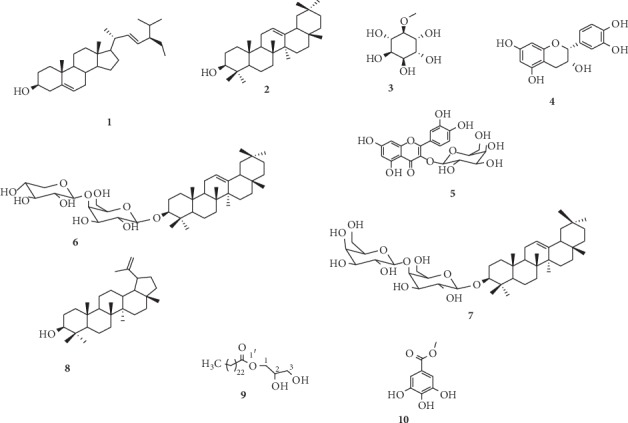
Chemical structures of compound isolated leaves and barks of *A. polyacantha. ***1**: stigmasterol, **2**: *β*-amyrin, **3**: 3-(O)-methyl-_D_-chiro-inositol, **4**: Epicatechin, **5**: Quercetin-3-(O)-galactoside, **6**: 3-(O)-[*β*-_D_-xylopyranosyl-(1 ⟶ 4)-*β*-_D_-galactopyranosyl]-oleanolic acid, **7**: 3-(O)-[*β*-_D_-galactopyranosyl-(1 ⟶ 4)-*β-*_D_-galactopyranosyl]-oleanolic acid, **8**: Lupeol, **9**: 2,3-dihydroxylpropyltétracosanoate, **10**: methyl gallate.

The chemical structures (Figure 1**)** and properties of compounds isolated from leaves and barks of*Acacia polyacantha* were determined using NMR (^1^H and ^13^C) data and in comparison with literature. These compounds were identified from barks as Lupeol C_30_H_50_O (**8;** Wwite crystal; melting point (m.p.): 220–222°C; [M]^+^ at *m*/*z* 426), 2,3-dihydroxypropyltetracosanoate C_27_H_54_O_4_ (**9;** white powder; m.p.: 86–88°C; [M+Na]^+^ at *m*/*z* 465), and methyl gallate C_8_H_8_O_5_ (**10;** white powder; m.p.: 197, 6–199°C; [M]^+^ at *m*/*z* 184), and from leaves as stigmasterol C_29_H_50_O (**1;** white powder, m.p.:134–135°C; *m*/*z* 414) [22], *β*-amyrinC_30_H_50_O (**2;** white powder, m.p.:187–190°C; *m*/*z* 426) [23], 3-*O*-méthyl-_D_-chiro-inositol C_7_H_14_O_6_ (**3;** white powder, m.p.:181°C; *m*/*z* 217; [*α*]_*D*_^25^: +60,00) [24], epicatechinC_15_H_14_O_6_ (**4;** red powder, m.p.:345–350°C; *m*/*z* 270) [25], quercetin-3-O-galactoside C_21_H_20_O_12_ (**5;** yellow powder, m.p.:230–232°C; *m*/*z* 464) [26], 3-*O*-[*β*-_D_-xylopyranosyl-(1 ⟶ 4)-*β*-_D_-galactopyranosyl]-oleanolic acid C_41_H_66_O_12_ (**6;** white powder, m.p.:1216–217°C; *m*/*z*773,4395 (cal. 773,4452 for C_41_H_66_O_12_Na+); [*α*]_*D*_^25^: +23,2° (c 1,25; MeOH)) [15], and 3-*O*-[*β*-galactopyranosyl-(1 ⟶ 4)-*β*-_D_-galactopyranosyl]-oleanolic acid C_42_H_68_O_13_ (**7;** white powder; *m*/*z* 803) [22]. These NMR (^1^H and ^13^C) spectra as well as main chemical shifts are presented in supplementary data ([Supplementary-material supplementary-material-1]).

### 3.2. Antibacterial Assay

The crude extracts, fractions, and compounds from *Acacia polyacantha* were tested for their antistaphylococcal activity against 14 staphylococcal bacterial strains, and the results are presented in Table 1. The results revealed that both leaf and bark extracts had inhibitory effects on 13/14 and 10/14 of the tested bacteria, respectively, with MICs ranging from 16 *μ*g/mL to 1024 *μ*g/mL as shown in Table 1. The activities of fractions were very selective but better than those of their extracts of origin. The MICs of fractions varied between 16 *μ*g/mL and 512 *μ*g/mL, with the activity of *F*1 and *F*3 obtained from barks being generally below 100 *μ*g/mL (Tables 2 and 3). The results obtained with the isolated compound also revealed selective and improved (smaller MICs) activities, which were in most cases ≤256 *μ*g/mL (Tables 1–3).

### 3.3. Effect of Efflux Pump Inhibitors on the Activities of Plant Samples

The extracts, selected fractions, and compounds were tested in the presence of two efflux pump inhibitors: chlorpromazine (CPZ) and carbonyl cyanide *m*-chlorophenylhydrazone (CCCP) for any improvement of their antistaphylococcal activities. The results obtained showed that, in the presence of CCCP as an efflux pump inhibitor, the activities of all samples were significantly improved; however, the degree of improvement varied from one bacterial strain to another (Table 4). Meanwhile, in the presence of CPZ as an efflux pump inhibitor, only the activity of compound **10** isolated from barks was improved on 10/14 bacterial strains tested (Table 5). After carrying out the previous test, the results revealed that compound **4** is the most active compound against the reference bacterial strain (ATCC 25923); this compound was selected and its effects on bacterial growth kinetics and proton-ATPase pumps were evaluated.

### 3.4. Effect on Growth Kinetics

**Figure 2:**
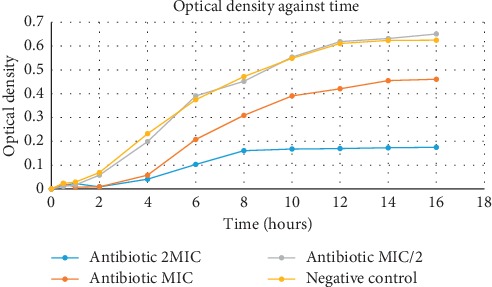
Effects of compound **4** on the growth kinetics of ATCC25923.

The results indicate that compound **4** affected the growth kinetics in a concentration-dependent manner (higher effects observed with increase concentration) and that this compound mainly acted by prolonging the lag phase from about 15 minutes in the negative control to about 2 hours in the case of the compound tested at concentrations of 0.5 × MIC, MIC, and 2 × MIC. The results are presented in Figure 2.

### 3.5. Effect on Proton-ATPase Pumps

**Figure 3:**
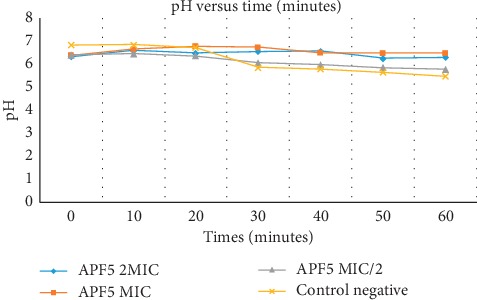
Effects of compound **4** on the proton-ATPase pumps.

In the absence of compound **4**, we observed rapid glucose-induced acidification of growth medium from the twentieth minute to the thirtieth minute, followed by slow acidification until the end of the experiment. Meanwhile, in the presence of compound **4**, at concentrations of MIC and 2 × MIC, this acidification was not observed; this is materialized by a linear tendency of the curves corresponding to these two concentrations contrary to the negative control curve which tends to decrease with time. These results are presented in Figure 3.

## 4. Discussion

### 4.1. Phytochemistry

Compounds isolated in this study included one sterol (stigmasterol), two triterpenes (*β*-amyrin and lupeol), two saponins (3-*O*-[*β*-D-galactopyranosyl-(1 ⟶ 4)-*β*-D-galactopyranosyl]-oleanolic acid, and 3-*O*-[*β*-D-xylopyranosyl-(1 ⟶ 4)-*β*-D-galactopyranosyl]-oleanolic acid), one sugar (3-*O*-methyl-D-chiro-inositol), and three flavonoids (epicatechin, quercetin-3-*O-*galactoside, and methyl gallate). Studies prior to this led to the isolation of the same compounds, as well as some which were not isolated in this study such as polyacanthoside A, from the leaves of *A. polyacantha* [11]. This difference in the number of compounds isolated could be explained by the fact that not only leaves but also stem barks were used in this study.

### 4.2. Antibacterial Assay

In the quest for new and efficient antistaphylococcal agents, attention must be paid to resistant strains as they are a major call for concern. Multidrug-resistant strains were used in this study, and in order to better appreciate the activities of plant samples, cutoff value established by Kuete [22] states that plant extracts are very active if their MICs are below 100 *μ*g/mL, moderately active if 100 ≤ MICs ≤ 625 *μ*g/mL, and weakly active if MICs >625 *μ*g/mL. On this basis, stem bark extract was very active against two bacteria strains SA01 and SA18 with MICs of 16 *μ*g/mL and 32 *μ*g/mL, respectively. The same observation was made for root extracts, however, with higher MIC of 64 *μ*g/mL against SA07 and SA 23. Leaf extract was very active against only one bacterium SA18 with an MIC of 16 *μ*g/mL. Apart from these cases, all extracts except for root extracts generally had moderate activity mostly ranging from 128 *μ*g/mL to 512 *μ*g/mL. It is worth noting that root extract presented a rather low activity generally with MICs ≥1024 *μ*g/mL against 9 of the tested bacteria strains. These activities are justified by the presence of secondary metabolites isolated in this study as well as those isolated from leaves of *A. polyacantha* in previous studies [23]. The activities of fractions were better compared to their extracts of origin. Among the fractions used in this study, three obtained from bark extracts were very active (MICs ≤ 100 *μ*g/mL) against 71.43 % (10/14) for *F*_1_, 57.14% (8/14) for *F*_10_, and 50% (7/14) for *F*_3_. Meanwhile, the rest as well as some of the fractions obtained from leaves presented a rather moderate activity (100 *μ*g/mL ≤ MIC ≥ 625 *μ*g/mL). Generally, this was the case with *F*_2_, *F*_4_, *F*_8_, and *F*_12_ all obtained from barks and *F*_9_′ obtained from leaves. It is worth noting that ethyl acetate fraction of roots, the *n*-butanol fraction of barks, and *F*_6_′, *F*_10_′, and *F*_11_′ all obtained from leaves had weak activity generally ≥1024 *μ*g/mL. The improvement of activity is in line with the result obtained by Mhalla et al. [27]. These results suggest that some compounds present in the extracts exerted antagonistic interaction and that fractionation effectively separated them, making those that had weak activity more active. According to the cutoff point established for pure compounds obtained from medicinal plants by [24], pure compounds are considered to have a significant activity if they have MIC <100 *μ*g/mL, moderate activity if 10 < MIC ≤ 100 *μ*g/mL, and low activity if they have MIC > 100 *μ*g/mL. On this basis, compound **3** (3-*O*-méthyl-_D_-chiro-inositol) and compound **4** (epicatechin) obtained from the leaves of *A. polyacantha* had moderate activity against 78.57 % (11/14) and 57.14 % (8/14) of the tested bacteria, respectively. The rest of the compounds isolated from *A. polyacantha* generally presented rather low activity. However, the MICs obtained with compound **2** (*β*-amyrin), compound **10** (methyl gallate), and compound **7**(3-*O*-[*β*-galactopyranosyl-(1 ⟶ 4)-*β*-_D_-galactopyranosyl]-oleanolic acid) were better (128 *μ*g/mL ≤ MIC ≤ 512 *μ*g/mL) compared to those obtained with compound **1** (stigmasterol), compound **5** (quercetin-3-*O*-glucoside), compound**6** (3-*O*-[*β*-_D_-xylopyranosyl-(1 ⟶ 4)-*β*-_D_-galactopyranosyl]-oleanolic acid) all obtained from leaves, and compound **8** (lupeol) and compound **9** (2,3-dihydroxypropyltetracosanoate) all isolated from barks. This result indicates that these compounds have the ability to inhibit staphylococcal growth. Their activities can be associated with the chemical structure; for example, compound 4 was isolated from a less active fraction suggesting that the presence of many hydroxyl groups engaged the compound in many interactions, thus impairing its real activity. However, after fractionation, these interactions could have been disengaged, thus permitting the compound to exert its real activity. Taking into consideration the classification established by Gatsing and Adoga, [25], an extract is said to have a bactericidal effect if the MBC/MIC ≤4 and bacteriostatic effect if MBC/MIC ˃ 4, and the observed effects were all bacteriostatic since this ratio was generally greater than 4. These results were corroborated by those of Esquenazi et al. [26] who have demonstrated in their work the activity of epicatechin against strains of *Staphylococcus aureus* and also those of Escandón et al. [28] who showed in their work the antibacterial activity of epicatechin against *Helicobacter pylori*.

### 4.3. Effect of Efflux Pumps Inhibitors on the Activities of Plant Samples

Efflux pumps play diverse roles in bacterial cells, one of which is to reduce the intracellular concentration of the exogenous substance (including antibiotics) which can act as their substrates, sending them out of the bacteria cells, thus, reducing the efficacy of these drugs. The improvement of the activity of all extracts, fractions, and compounds against tested bacterial strains in the presence of CCCP as efflux pump inhibitor, with activity improvement factors varying from 2 to 128 which are significant, suggests that, in the absence of EPI, the active substances are preferential substrates of the efflux pumps [23]. These results equally support the fact that the bacterial strains used in this study were multidrug-resistant strains overexpressing efflux pumps. However, the variation in percentage potentiation could be due to differences in the copy number of efflux pumps as shown in [29]. The activities of extracts, fractions, and compounds in the presence of chlorpromazine as an efflux pump inhibitor were rather reduced, and these results were not as expected [30]. This could be explained by the fact that the bacterial strains used in this study are Gram-positive strains which do not express RND [31] and that chlorpromazine reduces the permeability of the bacterial membrane to the sample [32].

### 4.4. Effect on Growth Kinetics

The growth kinetics of every bacterial cell is specific, and each phase reflects the biochemical processes taking place. The prolongation of any of these phases results from the inhibition of these biochemical processes, thus, inhibiting the growth of the bacteria in question. In this study, it was observed that compound **4** (Epicatechin) had inhibitory action at the lag phase; this was indicated by a prolonged lag phase at MIC and 2 × MIC. This prolongation of the lag phase could be explained by the presence of antibacterial molecule at an inhibitory concentration which inhibited the rapid biosynthesis of enzymes needed for metabolism and thus entry into the exponential phase [33]. Pharmacologically, this may limit the effects of the development of the bacterium in the colonization of the site of infection, which is usually the initial stage of infection.

### 4.5. Effect on Proton-ATPase Pumps

Proton-ATPase pumps play varying roles in bacteria cells; one of great importance is regulating the cytoplasm pH of *Staphylococcus*. Any inhibition of these pumps equally inhibits the growth of the bacteria [34]. The inhibition of these pumps by compound **4** (epicatechin) indicated by a constant pH of the culture medium when compound **4** was tested at MIC and at 2 × MIC indicates that these pumps are possible targets of the compound [35].

## 5. Limitations

As a limitation of the present investigation, the toxicity of this plant will further be performed to evaluate its safety.

## 6. Conclusion

This study evaluated the antistaphylococcal activity of extracts, fractions, and compounds from *Acacia polyacantha* leaves, stem barks, and roots. Epicatechin (compound **4)** acts at the latency phase and inhibits proton pumps; this led to the death of the bacterial cell. These results show that compound **4** (Epicatechin) from *Acacia polyacantha* has very good antistaphylococcal activity and would be a good candidate in the search for ways and means to combat diseases caused by sensitive and resistant *Staphylococci*.

## Figures and Tables

**Table 1:** Minimum inhibitory concentration (MIC) and minimum bactericidal concentration (MBC) in *μ*g/mL of crude extracts from *Acacia polyacantha* and ciprofloxacin against *S. aureus* strain.

Tested samples, MICs in *μ*g/mL, and MBCs in parentheses				
Bacteria	Stem bark extract	Leaf extract	Root extract	Ciprofloxacin
*ATCC25923*	256(-)	512(-)	1024(-)	8(-)
*MRSA 3*	256(-)	128(-)	128(-)	2(-)
*MRSA 4*	256(-)	128(-)	1024(-)	1
*MRSA 6*	1024(-)	256(-)	1024(-)	2
*MRSA 8*	512(-)	1024(-)	1024(-)	2(-)
*MRSA 9*	512(-)	512(-)	1024(-)	2(-)
*MRSA 11*	1024(-)	512(-)	1024(-)	2(16)
*MRSA 12*	1024(-)	512(-)	1024(-)	2(-)
*MSSA 01*	—	512(-)	1024(-)	2(-)
*SA 01*	16(-)	128(-)	512(-)	≤0.5(-)
*SA 07*		—	64(-)	≤0.5(-)
*SA 18*	32(-)	16(-)	512(-)	≤0.5(8)
*SA 23*	—	512(-)	64(-)	≤0.5(8)
*SA 88*	—	128(-)	—	≤0.5(-)

(-): ≥1024 *μ*g/mL; MIC: minimum inhibitory concentration; MBC: minimum bactericidal concentration; MRSA: methicillin-resistant *Staphylococcus aureus*; SA: *Staphylococcus aureus*; MSSA: methicillin susceptible *Staphylococcus aureus*.

**Table 2:** MICs (*μ*g/mL) of stem bark and leaf fractions.

Bacteria	Fractions, MICs, and MBCs (in parentheses) in *μ*g/mL										
	*F*_1_	*F*_2_	*F*_3_	*F*_4_	*F*_8_	*F*_10_	*F*_6_′	*F*_9_′	*F*_10_′	*F*_11_′	*F*_12_
*ATCC25923*	64(-)	—	16(-)	512(-)	256(-)	64(-)	—	512(-)	256(-)	—	512(-)
*MRSA 3*	32(-)	512(-)	64(-)	512(-)	256(-)	64(-)	—	256(-)	—	—	128(-)
*MRSA 4*	64(-)	—	64(-)	512(-)	512(-)	128(-)	—	—	—	—	—
*MRSA 6*	64(-)	256(-)	—	—	128(-)	64(-)	—	—	—	—	512(-)
*MRSA 8*	64(-)	—	32(-)	—	256(-)	64(-)	≤4(-)	512(-)	—	—	512(-)
*MRSA 9*	128(-)	—	64(-)	128(-)	128(-)	128(-)	—	256(-)	—	—	512(-)
*MRSA 11*	32(-)	512(-)	—	512(-)	128(-)	64(-)	—	512(-)	—	—	32(-)
*MRSA 12*	64(-)	256(-)	64(-)	—	256(-)	32(-)	—	512(-)	—	—	512(-)
*MSSA 01*	64(-)	256(-)	—	—	512(-)	64(-)	—	512(-)	—	—	128(-)
*SA 01*	—	—	64(-)	—	512(-)	—	—	512(-)	—	—	128(-)
*SA 07*	—	16(-)	—	—	256(-)	512(-)	—	256(-)	—	—	—
*SA 18*	512(-)	—	128(-)	—	128(-)	256(-)	—	256(-)	16(-)	—	16(-)
*SA 23*	64(-)	128(-)	—	256(-)	512(-)	64(-)	—	—	—	—	—
*SA 88*	64(-)	512(-)	256(-)	—	512(-)	256(-)	—	—	—	256	—

(-): ≥512 *μ*g/ml; MIC: minimum inhibitory concentration; MBC: minimum bactericidal concentration; MRSA: methicillin-resistant *Staphylococcus aureus*; SA: *Staphylococcus aureus*; MSSA: methicillin susceptible *Staphylococcus aureus*; *F*: fraction from leaves; *F*′: fractions from barks; fractions *F*_5_–*F*_7_, *F*_9_, *F*_1_′–*F*_5_′, *F*_7_′, *F*_8_′, and *F*_13_′ were not active.

**Table 3:** MICs (*μ*g/mL) of isolated compounds from leaves and stem barks of *Acacia polyacantha*.

Compounds, MICs, and MBCs (in parentheses) in *μ*g/mL								
Bacteria	**2**	**3**	**4**	**5**	**6**	**7**	**9**	**10**
*ATCC25923*	512 (-)	128 (-)	32	—	—	—	—	512 (-)
*MRSA 3*	256 (-)	32 (-)	16	—	—	256 (-)	—	256 (-)
*MRSA 4*	—	64 (-)	64	—	—	—	—	512 (-)
*MRSA 6*	—	64 (-)	32 (512)	—	—	128 (-)	—	256 (-)
*MRSA 8*	512 (-)	128 (-)	128	—	256 (-)	256 (-)	—	512 (-)
*MRSA 9*	256 (-)	64 (-)	32 (512)	—	—	64 (-)	—	512 (-)
*MRSA 11*	256 (-)	64 (-)	16 (512)	—	—	256 (-)	—	256 (-)
*MRSA 12*	256 (-)	128 (-)	32 (256)	—	—	512	—	512 (-)
*MSSA 01*	512 (-)	32 (-)	16	—	—	256 (-)	—	256 (-)
*SA 01*	128 (-)	128 (-)	64	512 (-)	—	256 (-)	512 (-)	32 (-)
*SA 07*	512 (-)	64 (-)	—	—	—	—	—	64 (-)
*SA 18*	—	64 (-)	64	512 (-)	—	512 (-)	512 (-)	16 (-)
*SA 23*	—	128 (-)	16	—	—	128 (-)	—	512 (-)
*SA 88*	256 (-)	512 (-)	—	—	—	—	—	128 (-)

(-): ≥512 *μ*g/mL; MIC: minimum inhibitory concentration; MBC: minimum bactericidal concentration; MRSA: methicillin-resistant *Staphylococcus aureus*; SA: *Staphylococcus aureus*; MSSA: methicillin susceptible *Staphylococcus aureus*; **2**: *β*-amyrin; **1**: stigmasterol; **3**: 3-*O*-méthyl-_D_-chiro-inositol; **4**: epicatechin; **5:** quercetin-3-*O-*glucoside; **6:** 3-*O*-[*β*-_D_-xylopyranosyl-(1 ⟶ 4)-*β*-_D_-galactopyranosyl]-oleanolic acid; **7**: 3-*O*-[*β* galactopyranosyl-(1 ⟶ 4)-*β*-D-galactopyranosyl]-oleanolic acid; **8**: Lupeol; **9**: 2,3-dihydroxypropyltetracosanoate; **10**: methyl gallate. Compounds **1** and **8** were not active on all tested bacteria.

**Table 4:** MICs (*μ*g/mL) of tested samples in the presence of carbonyl cyanide (*m*)-chlorophenylhydrazone (CCCP).

Bacteria	Tested samples, MIC (*μ*g/mL) in the presence of CCCP and AAF (in parentheses)									
	Bark crude extracts	Root extract	Leaf extract	Bark (EAF)	F8	3	4	7	10	Ciprofloxacin
*ATCC25923*	32 (**8**)	64 (**16**)	64 (**8**)	64 (**4**)	128 (**2**)	16 (**8**)	8 (**4**)	128 (≥**8**)	32 (**16**)	≤0.5 (≥1)
*MRSA 3*	≤4 (≥**64**)	≤4 (**32**)	≤4 (≥**32**)	64 (**2**)	≤2 (≥**128**)	≤2 (**≥16**)	≤2 (**≥8**)	≤4 (≥**64**)	8 (**32**)	≤0.5 (**≥4**)
*MRSA 4*	64 (**4**)	8 (**128**)	32 (**4**)	≤4 (≥**32**)	128 (**4**)	≤2 (**≥32**)	16 (**4**)	256 (≥**4**)	32 (**16**)	≤0.5 (**≥2**)
*MRSA 6*	16 (**64**)	256 (**4**)	≤4 (**≥64**)	256 (**2**)	64 (**2**)	≤2 (**≥32**)	32 (1)	8 (**16**)	16 (**16**)	≤0.5 (**≥4**)
*MRSA 8*	128 (**4**)	512 (**2**)	512 (**2**)	512 (≥**2**)	64 (**4**)	≤2 (**≥64**)	≤2 (**≥64**)	64 (**4**)	64 (**8**)	≤0.5 (**≥4**)
*MRSA 9*	16 (**32**)	512 (**2**)	128 **(4)**	16 (**16**)	64 (**2**)	4 (**16**)	2 (**16**)	16 (**4**)	16 (**32**)	≤0.5 (**≥4**)
*MRSA 11*	512 (**2**)	512 (**≥ 2**)	512 (1)	512 (1)	64 (**2**)	16 (**4**)	8 (**2**)	128 (**2**)	64 (**4**)	≤0.5 (**≥4**)
*MRSA 12*	512 (**2**)	512 (**≥ 2**)	512 (1)	- (-)	8 (**32**)	32 (**4**)	8 (**4**)	8 (**64**)	8 (**64**)	≤0.5 (**≥4**)
*MSSA 01*	32 (≥**32**)	128 (**8**)	256 (**2**)	8 (**4**)	32 (**16**)	16 (**2**)	8 (**2**)	64 (**4**)	8 (**32**)	≤0.5 (**≥4**)
*SA 01*	16 (**32**)	64 **(8)**	128 (1)	128 (1)	256 (**2**)	≤2 (**≥64**)	≤2 (**≥32**)	32 (**8**)	16 (**2**)	≤0.5 (≥1)
*SA 07*	512 (≥**2**)	≤4 (≥**16**)	512 (≥**2**)	64 (≥**16**)	128 (**2**)	16 (**4**)	- (≤1)	64 (≥**16**)	16 (**4**)	≤0.5 (≥1)
*SA 18*	16 (**2**)	8 (**64**)	≤4 (≥**4**)	≤4 (≥**32**)	64 (**2**)	≤2 (**≥32**)	16 (**4**)	512 (1)	≤2 (≥**8**)	≤0.5 (≥1)
*SA 23*	- (1)	512 (0.125)	64 (**8**)	≤4 (≥**128**)	128 (**4**)	16 (**16**)	- (-)	32 (**4**)	32 (**16**)	≤0.5 (≥1)
*SA 88*	128 (≥**128**)	128 (≥**8**)	64 (**2**)	256 (≥**4**)	32 (**16**)	32 (**16**)	32 (**≥32**)	128 (≥**8**)	128 (1)	≤0.5 (≥1)

(-): ≥1024 *μ*g/mL; CCCP: carbonyl cyanide m-chlorophenylhydrazone; AAF: activity amelioration factor; in bold are AAF ≥2; MIC: minimum inhibitory concentration; MBC: minimum bactericidal concentration; MRSA: methicillin-resistant *Staphylococcus aureus*; SA: *Staphylococcus aureus;* MSSA: methicillin susceptible *Staphylococcus aureus*; **3**: 3-*O*-méthyl-_D_-chiro-inositol; **4**: epicatechin; **7**: 3-*O*-[*β* galactopyranosyl-(1 ⟶ 4)-*β*-_D_-galactopyranosyl]-oleanolic acid; **10**: methyl gallate.

**Table 5:** MICs (*μ*g/mL) of tested samples in the presence of chlorpromazine (CPZ).

Bacteria	Tested samples, MIC (*μ*g/mL) in the presence of CPZ and AAFs (in parentheses)				
	Root extract	3	7	10	Ciprofloxacin
*ATCC25923*	- (≤1)	- (≤0.12)	- (nd)	64 (**8**)	≤0.5 (≥1)
*MRSA 3*	- (≤0.12)	- (≤0.03)	- (≤0.25)	64 (**4**)	≤0.5 (**≥4)**
*MRSA 4*	256 (**4**)	≤2 **(≥32)**	- (nd)	64 (**8**)	≥0.5 (**≥2)**
*MRSA 6*	- (≤1)	- (≤0.06)	- (≤0.12)	32 (**8**)	1 (**2**)
*MRSA 8*	- (≤1)	≤2 (**≥64)**	256 (1)	128 (**4**)	2 (1)
*MRSA 9*	- (≤1)	- (≤0.06)	- (≤0.06)	64 (**8**)	≤0.5 (**≥4)**
*MRSA 11*	- (nd)	64 (1)	- (≤0.25)	128 (**2**)	≤0.5 (**≥4)**
*MRSA 12*	- (nd)	- (≤0.12)	- (≤0.5)	64 (**8**)	2 (1)
*MSSA 01*	- (≤1)	- (≤0.03)	- (≤0.25)	64 (**4**)	0.5 (**4**)
*SA 01*	- (≤0.5)	- (≤0.12)	- (≤0.25)	64 (0.5)	≤0.5 (≥1)
*SA 07*	- (≤0.06)	- (≤0.06)	- (nd)	128 (0.5)	≤0.5 (≥1)
*SA 18*	- (≤0.5)	- (≤0.06)	- (≤0.5)	64 (0.25)	≤0.5 (≥1)
*SA 23*	- (≤0.06)	- (≤0.12)	- (≤0.12)	128 (**4**)	≤0.5 (≥1)
*SA 88*	- (nd)	- (≤0.5)	- (nd)	128 (1)	≤0.5 (≥1)

(-): ≥1024 *μ*g/mL; CCCP: carbonyl cyanide *m*-chlorophenylhydrazone; AAF: activity amelioration factor; in bold are AAF ≥2; MIC: minimum inhibitory concentration; MBC: minimum bactericidal concentration; MRSA: methicillin-resistant *Staphylococcus aureus*; SA: *Staphylococcus aureus*; MSSA: methicillin susceptible *Staphylococcus aureus*; bark and leaf extracts as well as fraction 8 and compound **4** were tested but were not active; nd: not determined.

## Data Availability

All data generated or analyzed during this study are included in this published article.

## Abbreviations

ATCC: American-type culture collection
ATP: Adenosine triphosphate
CCCP: Carbonyl cyanide *m*-chlorophenylhydrazone
CFU: Colony-forming unit
CIP: Ciprofloxacin
CPZ: Chlorpromazine
DMSO: Dimethyl sulfoxide
EPIs: Efflux pump inhibitors
INT: *p*-Iodonitrotetrazolium chloride
MBC: Minimal bactericidal concentration
MDR: Multidrug-resistant
MHA: Mueller–Hilton agar
MHB: Mueller–Hinton broth
MIC: Minimal inhibitory concentration
MRSA: Methicillin-resistant *Staphylococcus aureus*
MRSA: Methicillin-resistant *Staphylococcus aureus*
NMR: Nuclear magnetic resonance
RND: *Resistance nodulation-cell division*
SA: *Staphylococcus aureus*
TLC: Thin-layer chromatography.

## Supplementary Materials

Supplementary Materials RMN 1H, 13C, and major chemical shifts of studied compounds.
