# Supplementary material for: Antistaphylococcal Activity of Extracts, Fractions, and Compounds of Acacia polyacantha Wild (Fabaceae)
Source: Evid Based Complement Alternat Med. 2020 Mar 16;2020:2654247. doi: 10.1155/2020/2654247 (PMC7102469; doi:10.1155/2020/2654247)
Supplement: Supplementary Materials — RMN 1H, 13C, and major chemical shifts of studied compounds. [file 2654247.f1.doc]

Anti-staphylococcal activity of extracts, fractions and compounds of *Acacia polyacantha* wild (Fabaceae)

Fred A. Ashu^1.2^. Jean Na-Iya^3^. Brice E. N. Wamba^1^. Justin Kamga^3^. Paul Nayim^1^. Bathélémy Ngameni^4^. Veronique P. Beng^2^. Bonaventure T. Ngadjui^3^. Victor Kuete^1*^

*^1^Department of Biochemistry. Faculty of Science. University of Dschang. Dschang. Cameroon;*

*^2^Department of Biochemistry. Faculty of Science. University of Yaoundé I. Yaoundé. Cameroon;*

*^3^Department of Organic Chemistry. Faculty of Science. University of Yaoundé I. Yaoundé. Cameroon;*

*^4^Department of Pharmacognosy and Pharmaceutical Chemistry. Faculty of Medicine and Biomedical Science. University of Yaoundé I. Cameroon;*

*Author’s emails:*

*Fred A Ashu:* [*ashufred50@yahoo.com*](mailto:ashufred50@yahoo.com)*;*

*Jean Na-Iya:* [*jeannaiya2@gmail.com*](mailto:jeannaiya2@gmail.com)

*Brice E. N. Wamba:* [*wambaelvis@yahoo.fr*](mailto:wambaelvis@yahoo.fr)

*Justin Kamga:* [*kajuti55@yahoo.fr*](mailto:kajuti55@yahoo.fr)

*Paul Nayim:* [*nayimpaul@yahoo.fr*](mailto:nayimpaul@yahoo.fr)

*Bathélémy Ngameni:* [*bath_ngameni@yahoo.fr*](mailto:bath_ngameni@yahoo.fr)

*Veronique P. Beng:* [*v.penlap@yahoo.fr*](mailto:v.penlap@yahoo.fr)

*Bonaventure T. Ngadjui: ngadjuibt@yahoo.fr*

*Victor Kuete:* [*kuetevictor@yahoo.fr*](mailto:kuetevictor@yahoo.fr  )

**Corresponding author:**

**Tel: (+237) 677355927; E-mail:* [*kuetevictor@yahoo.fr*](mailto:kuetevictor@yahoo.fr)*; P.O. Box 1499 Bafoussam. Cameroon (Prof. Dr. Victor Kuete)*

***Compound 1***

**Stigmastérol** . C_29_H_48_O. White powder

RMN ^13^C (CDCl_3_. 150 MHz): 12.0 (C-_29_) ; 12.2 (C-_24_) ; 18.9 (C-_28_) ; 19.4 (C-_27_) ; 21.1 (C-_26_) ; 21.4 (C-_11_) ; 22.7 (C-_22_) ; 24.4 (C-_15_) ; 25.4 (C-_23_) ; 29.4 (C-_16_) ; 29.7 (C-_25_) ; 31.7 (C-_7_ et C-_8_) ; 31.9 (C-_2_) ; 36.5 (C-_10_) ; 37.2 (C-_1_) ; 39.7 (C-_12_) ; 40.6 (C-_18_) ; 42.2 (C-_13_) ; 42.3 (C-_4_) ; 50.2 (C-_22_) ; 51.2 (C-_9_) ; 55.9 (C-_17_) ; 56.8 (C-_14_) ; 71.8 (C-_3_) ; 121.7 (C-_6_) ; 129.3 (C-_21_) ; 138.3 (C-_20_) ; 140.7 (C-_5_) [1].

**RMN ^13^C (CDCl_3_. 150 MHz) of compound 1**

**RMN ^1^H (CDCl_3_. 600 MHz) of compound 1**

***Compound 2***

**β-amyrine.** C_30_H_50_O. White powder

RMN ^13^C (CDCl_3_. 150 MHz): 15.5 (C-25) ; 15.6 (C-24) ; 16.3 (C-26) ; 18.6 (C-6) ; 23.6 (C-11) ; 23.7 (C-30) ; 25.9 (C-27) ; 26.1(C-16) ; 26.2 (C-15) ; 26.6 (C-15) ; 27.2 (C-27) ; 28.0 (C-23) ; 28.4 (C-28) ; 31.0 (C-20) ; 32.5 (C-7) ; 32.6 (C-17) ; 33.8 (C-29) ; 34.7 (C-21) ; 36.9 (C-10) ; 37.1 (C-22) ; 38.6 (C-4) ; 38.8 (C-1) ; 39.8 (C-8) ; 41.7 (C-14) ; 46.8 (C-19) ; 47.2 (C-18) ; 47.6 (C-9) ; 55.2 (C-5) ; 79.0 (C-3) ; 121.7 (C-12) ; 145.2 (C-13) [2].

**RMN ^13^C (CDCl_3_. 150 MHz) of compound 2**

**RMN ^1^H (CDCl_3_. 600 MHz) of compound 2**

***Compound 3***

**3-*O*-méthyl-D-Chiro-inositol;** C_7_H_14_O_6_**.** White powder

RMN ^13^C (D_2_O. 150 MHz): 59.6 (MeO-) ; 69.7 (C-_5_) ; 70.5 (C-_2_) ; 71.4 (C-_6_) ; 71.6 (C-_1_) ; 72.0 (C-_4_) ; 82.7 (C-_3_) [3].

**RMN ^1^H (D_2_O. 150 MHz) of compound 3**

**RMN ^1^H (D_2_O. 600 MHz) of compound 3**

***Compound 4***

**Epicatechin;** C_15_H_14_O_6_ . Red powder

RMN ^13^C (DMSO-*d_6_*. 150 MHz): 28.6(C-_4_) ; 65.3(C-_3_) ; 78.5(C-_2_) ; 94.5(C-_8_) ; 95.5 (C-_6_) ; 98.9 (C-_4a_) ; 115.2 (C-_2’_) ; 115.3 (C-_5’_) ; 118.4 (C-_6’_) ; 131.0 (C-_1’_) ; 144.8 (C-_4’_) ; 144.9 (C-_3’_) ; 156.1 (C-_8a_) ; 156.6 (C-_5_) ; 156.9 (C-_7_) [4].

**RMN ^1^H (DMSO-*d_6_*. 150 MHz) of compound 4**

**RMN ^1^H (DMSO-*d_6_*. 600 MHz) of compound 4**

***Compound 5***

**quercetin-3-O-galactoside**. C_21_H_20_O_12_. Yellow powder

RMN ^13^C (DMSO-*d_6_*. 150 MHz): 60.6 (C-_6”_) ; 68.5 (C-_4”_); 71.5 (C-_2”_); 73.6 (C-_3”_); 76.3 (C-_5”_); 93.4 (C-_8_); 99.1 (C-_6_) ; 102.2 (C-_1”_);104.2 (C-_4a_) ; 115.6 (C-_2’_) ; 116.3 (C-_5’_); 121.5(C-_1’_) ; 122.4 (C-_6’_) ; 133.82 (C-_3_); 145.3 (C-_3’_) ; 148.9 (C-_4’_) ; 156.6 (C-_8a_) ; 156.7 (C-_2_) ; 161.6 (C-_5_) ; 164.7 (C-_7_) ; 177.7 (C-_4_) [5].

**RMN ^13^C (DMSO-*d_6_*. 150 MHz) of compound 5**

**RMN ^1^H (DMSO-*d_6_*. 600 MHz) of compound 5**

***Compound 6***

**3-O-[β-D-xylopyranosyl-(1→4)-β-D-galactopyranosyl]-oleanolic acid;** C_41_H_66_O_12_ . White powder

RMN ^13^C (CD_3_OD. 150 MHz): 14.5 (C-_25_) ; 15.6 (C-_24_) ; 16.3 (C-_26_) ; 17.9 (C-_6_) ; 22.6 (C-_30_) ; 22.7 (C-_16_) ; 23.1 (C-_11_) ; 25.0 (C-_27_) ; 25.6 (C-_2_) ; 27.1 (C-_23_) ; 27.4 (C-_15_) ; 30.2 (C-_20_) ; 32.2 (C-_29_) ; 32.4 (C-_22_) ; 32.6 (C-_7_) ; 33.5 (C-_21_) ; 36.5 (C-_10_) ; 38.4 (C-_1_) ; 38.7 (C-_4_) ; 39.2 (C-_18_) ; 41.3 (C-_8_) ; 41.5 (C-_14_) ; 45.8 (C-_19_) ; 46.2 (C-_17_) ; 47.6 (C-_9_) ; 55.6 (C-_5_) ; 60.6 (C-_6’_) ; 65.7 (C-_5’’_) ; 69.5 (C-_4’’_) ; 73.5 (C-_2’’_) ; 74.0 (C-_2’_) ; 74.6 (C-_5’_) ; 74.9 (C-_3’_) ; 76.4 (C-_3’’_) ; 79.5 (C-_4’_) ; 89.5 (C-_3_) ; 104.0 (C-_1’’_) ; 105.1 (C-_1’_) ; 122.2 (C-_12_) ; 143.8 (C-_13_) ; 180.5 (C-_28_) [6].

**RMN ^13^C (CD_3_OD. 600 MHz) of compound 6**

**RMN ^1^H (CD_3_OD. 600 MHz) of compound 6**

***Compound 7***

**3-O-[β-galactopyranosyl-(1→4)-β-D-galactopyranosyl]-oleanolic acid;** C_42_H_68_O_13_. White powder

RMN ^13^C (CD_3_OD. 150 MHz): 14.5 (C-_25_) ; 15.5 (C-_24_) ; 16.3 (C-_26_) ; 18.0 (C-_6_) ; 22.7 (C-_30_) ; 23.1 (C-_16_) ; 25.0 (C-_11_) ; 25.7 (C-_27_) ; 27.1 (C-_2_ et C-_23_) ; 27.4 (C-_15_) ; 32.2 (C-_20_) ; 32.4 (C-_7_) ; 32.6 (C-_22_ et C-_29_) ; 33.5 (C-_21_) ; 36.5 (C-_10_) ; 38.4 (C-_4_) ; 39.2 (C-_1_) ; 39.0 (C-_8_) ; 41.4 (C-_18_) ; 41.5 (C-_14_) ; 45.9 (C-_17_) ; 45.7 (C-_9_) ; 46.3 (C-_19_) ; 55.6 (C-_5_) ; 61.7 (C-_6’_) ; 70.1 (C-_4’’_) ; 70.4 (C-_2’’_) ; 74.9 (C-_3’_) ; 76.2 (C-_3’’_) ; 76.5 (C-_2’_) ; 77.0 (C-_5’_) ; 77.1 (C-_5’’_) ; 79.7 (C-_4’_) ; 90.1 (C-_3_) ; 103.1 (C-_1’_) ; 104.0 (C-_1’’_) ; 122.2 (C-_12_) ; 143.8 (C-_13_) ; 180.7 (C-_28_) [7].


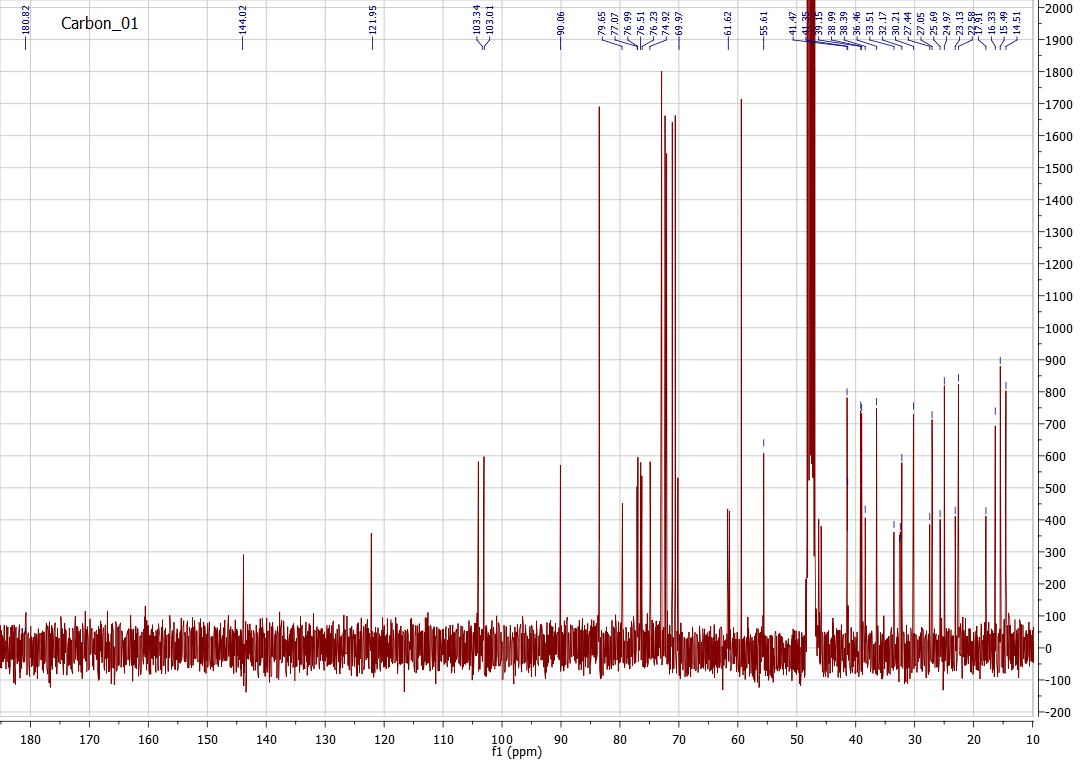


**RMN ^13^C (CD_3_OD. 600 MHz) of compound 7**


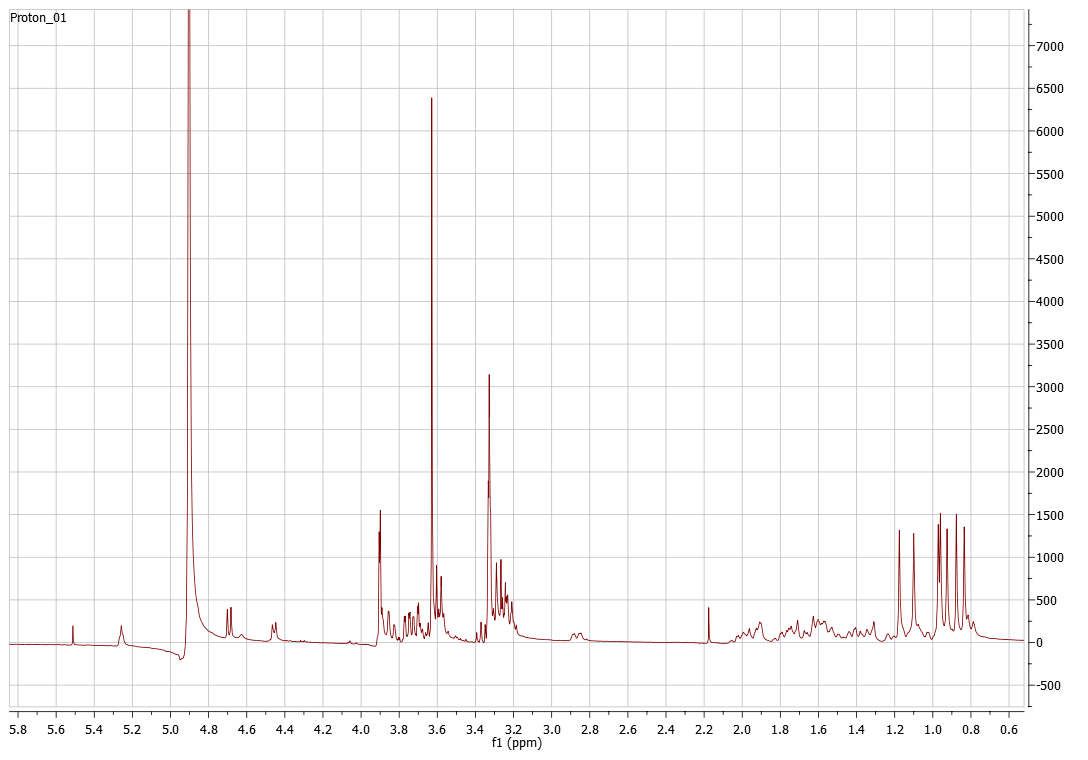


**RMN ^1^H (CCl_3_OD. 600 MHz) of compound 7**

***Compound 8***

**Lupeol.** C_30_H_50_O. White crystals

RMN ^13^C(CDCl_3_. 150 MHz): 14.5 (C-_27_). 15.3 (C-_24_). 15.9 (C-_25_). 16.1 (C-_26_). 18.0 (C-_28_). 18.3 (C-_6_). 19.3 (C-_30_). 20.9 (C-_11_). 25.1 (C-_12_). 27.4 ( C-_2_ et C-_15_). 28.0 (C-_23_). 29.7 (C-_21_). 34.3 (C-_7_). 35.6 (C-_16_). 37.1 (C-_10_). 38.0 (C-_13_). 38.7 (C-_1_). 40.8 (C-_8_). 48.0 (C-_18_). 50.4 (C-_9_). 55.3 (C-_5_). 79.0 (C-_3_). 109.3 (C-_29_). 150.9 (C-_20_) [8].


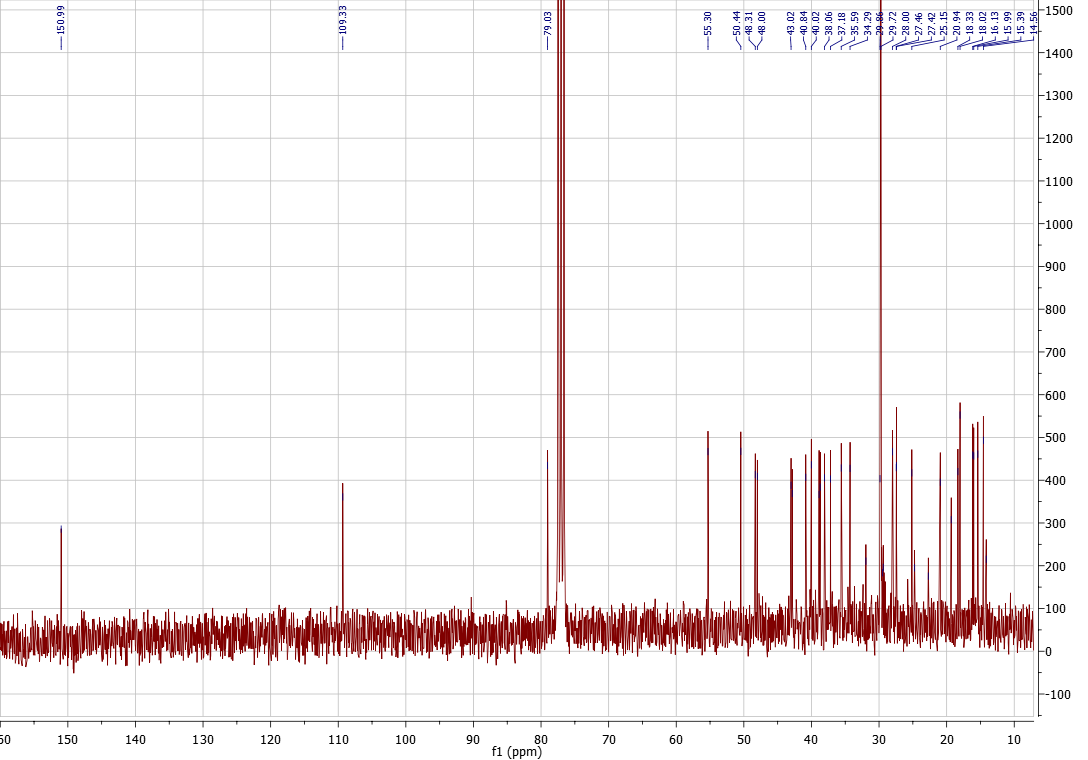


**RMN ^13^C (CD_3_Cl. 150 MHz) of compound 8**


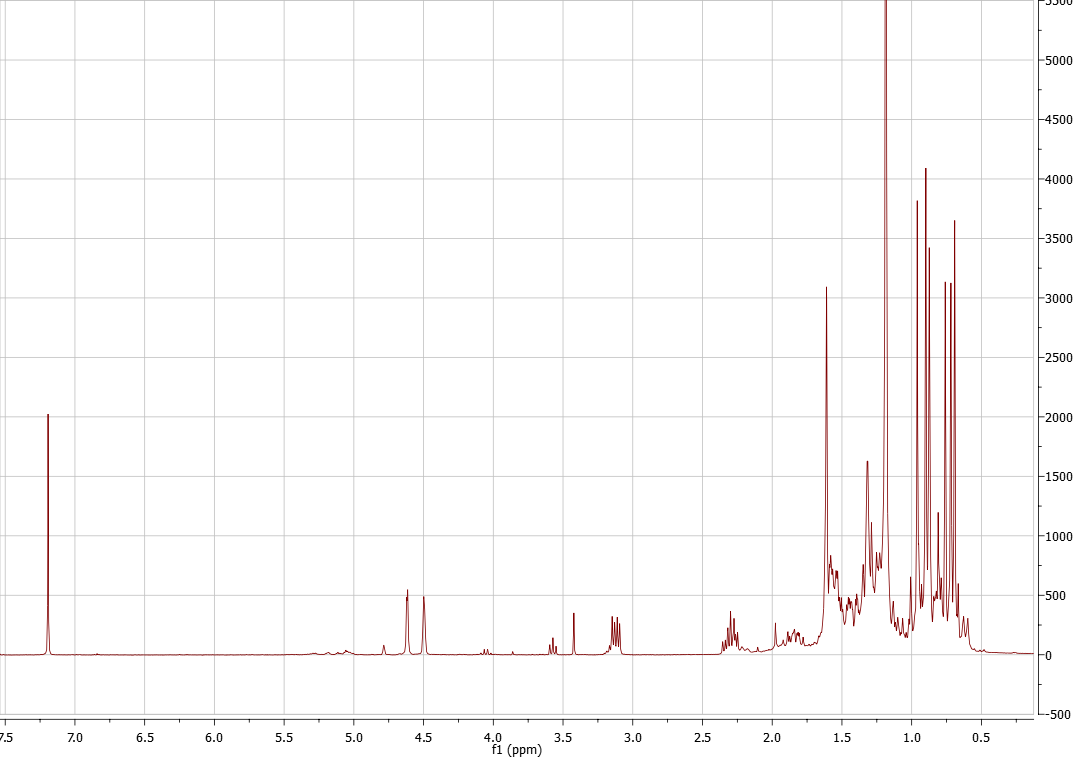


**RMN ^1^H (CD_3_Cl. 600 MHz) of compound 8**

***Compound 9***

**2.3-dihydroxypropyltetracosanoate.**  C_27_H_54_O_4_ (mp 86-88°C. m/z 465); White powder

RMN ^13^C (DMSO-*d_6_*. 150 MHz): 17.7 (C-_24’_) ; 26.5 (C-_23’_) ; 28.3 (C-_3’_) ; 30.1-33.5 (C-_4’_-C-_21’_) ; 35.4 (C-_22’_); 37.9 (C-_2’_) ; 67.0 (C-_3_) ; 68.1 (C-_1_) ; 73.8 (C-_2_) ; 178.2 (C-_1’_) [9].

**RMN ^13^C (DMSO-d_6_. 150 MHz) of compound 9**

**RMN ^1^H (CD_3_Cl. 600 MHz) of compound 9**

***Compound 10***

**Méthylgallate ;** C_8_H_8_O_5_. White powder;

RMN ^13^C (CD_3_OD. 150 MHz): 50.9 (Me-O) ; 108.6 (C-_2_ et C-_6_) ; 120.0 (C-_1_) ; 138.3 (C-_4_) ; 145.0 (C-_3_ et C-_5_) ; 167.6 (O=C) [9].

**RMN ^13^C (CD_3_OD. 150 MHz) of compound 10**

**RMN ^1^H (CD_3_OD. 600 MHz) of compound 10**

***REFERENCES***

[1] V. S. P. Chaturvedula. I. Prakash. “Isolation of stigmasterol and β-Sitosterol from the dichloromethane extract of *Rubus suavissimus.*” *International Current Pharmaceutical Journal*. 1: 239-242. 2012.

[2] S. B. Mahato. A. P Kundu. “^13^C NMR spectra of pentacyclic triterpenoids: A compilation and some salient features.” *Phytochemistry*. 37. 1517-1575. 1994.

[3] N. Sharma. M. K. Verma. D. K. Gupta. N. K. Satti. R. K. Khajuria. “Isolation and quantification of D-pinitol in *Argyrolobium roseum* *plant*. by 1H-NMR.” *Journal of Saudi Chemical Society*. 20: 81–87. 2016.

[4] I. K. Adnyana. Y. Tezuka. S. Awale. A. H. Banskota. K. Q. Tran. S. Kadota. “Quadranosides VI-XI. six new triterpene glucosides from the seeds of *Combretum quadrangulare*.” Chem. Pharm. Bull.. 48(8): 1114-1120. 2000.

[5] L. W. Xu. J. Chen. H. Y. Qi. Y. P. Shi. “Phytochemicals and their biological activities of plants in *Tagetes* L.” *Chinese Herbal Medicines*. 4 (2): 103-117. 2012.

[6] G. W. Fotso. J. Na-Iya. A. T. Mbaveng. P. A. Yves. I. Demirtas. V. Kuete. Y. Samuei. B. Ngameni. T. Efferth. B. T. Ngadjui. “Polyacanthoside A. a new oleanane-type triterpenoid saponin with cytotoxic effects from the leaves of *Acacia polyacantha* (Fabaceae)” *Natural Product Research*. 1-6. doi: 10.1080/14786419.2018.1486312. 2018.

[7] V. S. P. Chaturvedula. J. K. Schilling. J. S. Miller. R. Andreantsiferana. V. E. Rasamison. D. G. I. Kingston. “New cytotoxic oleanane saponins from the infrutescences of Polyscias amplifolia.” *Planta medica*. 69:440–449. 2003.

[8] G. W. Fotso. A. N. Ntumy. E. Ngachussi. M. Dube. R. Mapitse. G. D. W. F. Kapche. K. Andrae-Marobela. B. T. Ngadjui. B. M. Abegaz. Epunctanone. a new benzophenone and other secondary metabolites from *Garcinia epunctata* Stapf. (Guttiferae).” *Helv Chim Acta*. 97: 957-964. 2014.

[9] H. J. Lee. M. Khan. H. Y. Kang. D. H. Choi. P. Mi-Jin. L. Hyun-Jung. “Rare natural products from the wood of *Magnolia grandiflora.”* *Chemistry Natutral Compdounds*. 46: 243-244. 2000.

[10] J. G. Choi. O. H. Kang. Y. S. Lee. Y. C. Oh. H. S. Chae. H. J. Jang. D. W. Shin. D. Y. Kwon. “Antibacterial activity of methyl gallate isolated from galla rhois or carvacrol combined with nalidixic acid against nalidixic acid resistant bacteria” Molecules. 14: 1773-1780. doi:10.3390/molecules14051773. 2009.
